# Supplementary material for: Factors affecting chronic low back pain among high school baseball players in Japan: A pilot study
Source: PLoS One. 2023 Jan 26;18(1):e0280453. doi: 10.1371/journal.pone.0280453 (PMC9879397; doi:10.1371/journal.pone.0280453)
Supplement: Attachment — Submitted filename: plose.docx [file pone.0280453.s001.docx]

Top of Form

|  |  |  |
| --- | --- | --- |

|  | Authors did a study on **Factors Affecting Chronic Low Back Pain Among High School Baseball Players in Japan: A Pilot Study**.  However, I think the study contains many factual errors and needs to be substantially revised to be considered for publication.   The first concern I have, regards the language use. I think the study needs substantial English language editing to make it more clear for the readers.  Secondly, the authors need to tell us about the importance of their study and findings they reached.  Introduction  The authors start introduction with the sentence IN LINE 45 : „ whereas chronic LBP (CLBP), defined as LBP lasting at least three months, is observed in 1–40% of all baseball players.“ it is needed to be rechecked.  Some sentences need to be more clear for the reader such as in line 51: In addition, reports have identified psychosocial factors and central sensitization as risk factors for CLBP [3, 4]; what does mean…  Sometimes authors present us with statements that need to be followed with references, but the references are omitted. For example, on page …:  The mental aspects of chronic pain  50 include depression, anxiety, catastrophizing, kinesiophobia, and a decline in self efficacy. What literature are the authors referring to?  The introduction section more or less provides hints to the relevant background but **I cannot see how the experimental questions can be drawn from the given information**. For instance, in line 59 the statement of the problem has finished with this sentence, Preventing or rehabilitating LBP in high school players may reduce LBP prevalence among college and professional athletes”  The hypotheses cannot be drawn from the information provided? I think it is needed to be revised in a standard manner, because it is not the aim of the study.    METHODS  We see very important inconsistency: in the Abstract the authors mentioned In  the results section, authors reported many p values! That is far too many for the scope of this study and the authors almost certainly committed type 1 error, as the number of p values is as big as the sample.  What grip strength was measured? Is it relevant to the aim of the study?  What scale did select to measure functional tests in this study? Does “performed or not performed” is a suitable scale to measure this variable?  Is the level and history of athletes considered in sample selection? How authors control this variable?  Althoutgh, this investigation is a poilot, but the sample size is needed to to be sufficient for this research based on power, alpha level and ….? Is the sample size sufficient for this research?  In the manuscript, which results support the discussion on “Our study suggested that kinesiophobia is a significant cause of CLBP among…”  Was this study a cause-effect study or not?  With regards to results, kinesiophobia is the most important factor in CLBP? What about other measured variables?    I also recommend a language check by a native speaker since there are errors all over the text |
| --- | --- |
|  |  |
|  |  |

Bottom of Form
