# Supplementary material for: Factors affecting chronic low back pain among high school baseball players in Japan: A pilot study
Source: PLoS One. 2023 Jan 26;18(1):e0280453. doi: 10.1371/journal.pone.0280453 (PMC9879397; doi:10.1371/journal.pone.0280453)
Supplement: Attachment — Submitted filename: Responce to Reviewers.docx [file pone.0280453.s002.docx]

June 28, 2022

Professor Dr Matias Noll

Academic Editor

*PLOS ONE*

Dear Professor:

**Revised manuscript ID PONE-D-21-25318**

**Title: Factors affecting chronic low back pain among high school baseball players in Japan: a pilot study**

We are grateful for the opportunity to revise our manuscript and for the helpful comments from the reviewers. The comments were highly insightful and helped improve the quality of our paper. Please find below the point-by-point responses to the reviewers’ comments.

We hope that our revisions have made the manuscript suitable for publication in *PLOS ONE*

Sincerely,

Hidetoshi Nakao

Faculty of Social Work Studies

Department of Physical Therapy Josai International University 1 Gumyo, Togane City

Chiba 283-8555, Japan

Tel.: +81-475-55-8800

Fax: +81-475-55-8811

Email: [h_nakao@jiu.ac.jp](mailto:h_nakao@jiu.ac.jp)

Authors did a study on **Factors Affecting Chronic Low Back Pain Among High School Baseball Players in Japan: A Pilot Study**.

**Comments from Reviewer 1:**

However, I think the study contains many factual errors and needs to be substantially revised to be considered for publication.

The first concern I have, regards the language use. I think the study needs substantial English language editing to make it more clear for the readers. Secondly, the authors need to tell us about the importance of their study and findings they reached.
Introduction

**Response:**

**Thank you for the insightful comments. The revised manuscript has been edited for English language by Editage, a professional English language editing service. I have attached the proofreading certificate at the end of this document. Furthermore, significant changes have been made in the revised manuscript (revised texts are represented in red) to highlight the importance of the study and its conclusions.**

The authors start introduction with the sentence IN LINE 45 : „ whereas chronic LBP (CLBP), defined as LBP lasting at least three months, is observed in 1–40% of all baseball players.“ it is needed to be rechecked.

**Response:**

**Thank you for your helpful comment. The age range of baseball players with CLBP is wide. In the revised manuscript, we have categorized LBP prevalence according to age groups.**

**The revised text is as follows (Lines 47–51, page 4):**

LBP occurs not only in adulthood but also among young athletes [1]. The LBP prevalence among young baseball players aged 12−15.5 ranges from 8.3−15% [2]. Furthermore, LBP affects up to 48% of Japanese college baseball players [3], and the prevalence is also high in older baseball players.

Some sentences need to be more clear for the reader such as in line 51: In addition, reports have identified psychosocial factors and central sensitization as risk factors for CLBP [3, 4]; what does mean…

Sometimes authors present us with statements that need to be followed with references, but the references are omitted. For example, on page …:
The mental aspects of chronic pain

50 include depression, anxiety, catastrophizing, kinesiophobia, and a decline in self efficacy. What literature are the authors referring to?

**Response:**

**Thank you for the comment. The text has been edited to improve clarity in the revised manuscript. Furthermore, we have included citations in the texts.**

**The revised text is as follows (Lines 64–68, page 5):**

Chronic pain constitutes both physical and mental aspects. The mental aspects include depression, anxiety, catastrophizing, kinesiophobia, and a decline in self-efficacy [10-14]. In addition, reports have identified psychosocial factors and central sensitization as risk factors for CLBP [15, 16]. Psychosocial factors may hinder athletes from resuming the sport and affect their performance.

The introduction section more or less provides hints to the relevant background but **I cannot see how the experimental questions can be drawn from the given information**. For instance, in line 59 the statement of the problem has finished with this sentence, Preventing or rehabilitating LBP in high school players may reduce LBP prevalence among college and professional athletes”

The hypotheses cannot be drawn from the information provided? I think it is needed to be revised in a standard manner, because it is not the aim of the study.

**Response:**

**Thank you for the insightful comment. We agree with your observation. Hence, in the revised manuscript, we have included a paragraph that illustrates the hypothesis and motivation behind this study. Although we have revised the entire Introduction section, the purpose of the study is mentioned in the last paragraph of this section.**

**The revised text is as follows (Lines 69–73 and page 5):**

To the best of our knowledge, no study has examined the association of body compositions with CLBP intensity in high school baseball players. Hence, this study aimed to examine factors, such as physical composition, pain evaluation, and load related to LBP, that contribute to CLBP among high school baseball players. We also aimed to identify factors that promote and alleviate CLBP.

METHODS
We see very important inconsistency: in the Abstract the authors mentioned In  the results section, authors reported many p values! That is far too many for the scope of this study and the authors almost certainly committed type 1 error, as the number of p values is as big as the sample.

**Response:**

**Thank you for the comment. We agree with you. We have reduced the number of reported p values, as this study aimed to assess physical compositions and pain intensity associated with CLBP, and identify baseball-related loads associated with CLBP. The main objective of this study is included in the last paragraph of the Introduction section of the revised manuscript.**

**The revised text is as follows (Line number 69–73 and page number 5):**

To the best of our knowledge, no study has examined the association of body compositions with CLBP intensity in high school baseball players. Hence, this study aimed to examine factors, such as physical composition, pain evaluation, and load related to LBP, that contribute to CLBP among high school baseball players. We also aimed to identify factors that promote and alleviate CLBP.

What grip strength was measured? Is it relevant to the aim of the study?

**Response:**

**Thank you for the question. We agree that there is no relationship between grip strength and LBP. Therefore, we have removed all information on grip strength in the revised manuscript.**

What scale did select to measure functional tests in this study? Does “performed or not performed” is a suitable scale to measure this variable?

**Response:**

**Thank you for the comment. In the revised manuscript, we have removed the functional test because it was not as important as the other variables.**

Is the level and history of athletes considered in sample selection? How authors control this variable?

**Response:**

**Thank you for the question. Table 1 presents the years of experience and position of the athletes at baseline and at the onset of LBP. The number of years of experience of players in each group was similar. We recognize that there were many pitchers in the CLBP group; however, since the players’ positions were diverse, we opine that it will be the participants of analysis as the number of data increases in the future. The players studied were all baseball players from the same high school; therefore, we added the amount and frequency of practice of the participants as follows.**

**The revised text is as follows (Lines 85–89, page 6):**

The practice frequency of the players was 6 days a week with an average of 4–5 hours per day. Japanese high school baseball games are conducted during the off-season period between December and March, and there are no external games. The practice activities during this period are running, defensive and batting, and strength training. The number of pitching practices decreased at this time.

Althoutgh, this investigation is a poilot, but the sample size is needed to to be sufficient for this research based on power, alpha level and ….? Is the sample size sufficient for this research?

**Response:**

**Thank you for the comment. We increased the number of participants during the recruitment period. Although the number of participants with CLBP did not change, we calculated the effective sample size using the TSK value as an example using the SPSS software. We also provided a reference on the valid number of samples in a pilot study (lines 141–149, pages 9–10).**

In the manuscript, which results support the discussion on “Our study suggested that kinesiophobia is a significant cause of CLBP among…”

Was this study a cause-effect study or not?

With regards to results, kinesiophobia is the most important factor in CLBP? What about other measured variables?

**Response:**

**Thank you for the comment. We observed that TSK showed an increase in the longitudinal comparison of athletes with CLBP. Other variables either remained the same or reduced, making TSK a CLBP-related factor. Therefore, we examined related factor for longitudinal changes rather than cause-effect.**

I also recommend a language check by a native speaker since there are errors all over the text

**Response:**

**As per your suggestion, we have submitted our manuscript to Editage for professional English language editing.**
